# Effects of Repetitive Transcranial Magnetic Stimulation on the Primary Motor Cortex of Individuals with Fibromyalgia: A Systematic Review and Meta-Analysis

**DOI:** 10.3390/brainsci12050570

**Published:** 2022-04-28

**Authors:** Hyunjoong Kim, Jihye Jung, Sungeon Park, Younglan Joo, Sangbong Lee, Seungwon Lee

**Affiliations:** 1 Department of Physical Therapy, Graduate School, Sahmyook University, 815, Hwarang-ro, Seoul 01795, Korea; doong18324@gmail.com (H.K.); spring1056@naver.com (S.P.); jooyounglan87@gmail.com (Y.J.); leesb109@naver.com (S.L.); 2 Institute of SMART Rehabilitation, Sahmyook University, 815, Hwarang-ro, Seoul 01795, Korea; jihye3752@gmail.com; 3 Department of Physical Therapy, Sahmyook University, 815, Hwarang-ro, Seoul 01795, Korea

**Keywords:** repetitive transcranial magnetic stimulation, fibromyalgia, primary motor cortex, psychosocial factor

## Abstract

The purpose of this study was to quantify the effect of repetitive transcranial magnetic stimulation (rTMS), which is recommended for the improvement of some pain-related symptoms and for antidepressant treatment, on the primary motor cortex (M1) in patients with fibromyalgia (FM). We searched for studies comparing rTMS and sham rTMS in the M1 of FM patients. Pain intensity, quality of life, health status, and depression were compared with or without rTMS for at least 10 sessions. We searched four databases. Quality assessment and quantitative analysis were performed using RevMan 5.4. After screening, five randomized controlled trials of 170 patients with FM were included in the analysis. As a result of the meta-analysis of rTMS on the M1 of individuals with FM, high-frequency rTMS resulted in a significant improvement on quality of life (MD = −2.50; 95% CI: −3.99 to −1.01) compared with sham rTMS. On the other hand, low-frequency rTMS resulted in a significant improvement on health status (MD = 15.02; 95% CI: 5.59 to 24.45). The application of rTMS to the M1 is proposed as an adjunctive measure in the treatment of individuals with FM. Because rTMS has various effects depending on each application site, it is necessary to classify sites or set frequencies as variables.

## 1. Introduction

Fibromyalgia (FM) is a syndrome characterized by musculoskeletal pain, fatigue, and tender points [1]. The cause of FM is not clearly known [2] as it is a diffuse, nonfocal chronic pain condition that occurs in 2–4% of the population [3]. For FM, specialists say that it is a disease associated with central sensitization due to dysfunction of the nociceptive nervous system [4]. Therefore, in a review study of factors that may contribute to the pathophysiology of FM, exposure to psychosocial stressors as well as physical factors may have an influence [5].

Repetitive transcranial magnetic stimulation (rTMS) is a noninvasive brain stimulation (NIBS) method that can modulate cerebral cortex regions using electromagnetic fields [6,7,8]. In FM patients, rTMS is known to have analgesic effects in the pain-related limbic area and the descending pain pathway [9,10]. In a related meta-analysis, stimulation of the primary motor cortex (M1) with rTMS was effective for pain control in chronic pain and fibromyalgia [9,11,12]. However, FM patients with pain should consider not only physical factors but also psychosocial factors. Evidence-based guidelines for rTMS suggest that only stimulation of the dorsal prefrontal cortex (DLPFC) has antidepressant effects on psychosocial factors [6]. The use of rTMS as a therapeutic antidepressant treatment was approved by the FDA in 2008. In addition, lasting effects remain when rTMS is applied to the left prefrontal area [13]. Nevertheless, no conclusive evidence has been found that stimulation of the M1, which is the most common stimulation site for chronic pain, is effective for antidepressant effects [12].

Therefore, in our review, studies that measured the effects of rTMS on pain and psychosocial factors on the M1 of individuals with FM in randomized controlled trials (RCTs) were synthesized through quantitative and qualitative analysis. 

## 2. Materials and Methods

### 2.1. Study Design

This study was a systematic review and meta-analysis that was created to integrate and analyze rTMS studies conducted on patients with FM. This study followed the Preferred Reporting Items for Systematic Reviews and Meta-Analysis (PRISMA) guidelines. The protocol used in this study was registered in the International Prospective Register of Systematic Reviews (PROSPERO) (No.: CRD42021226173).

### 2.2. Search Strategy and Selection of Studies

#### 2.2.1. Inclusion Criteria

1. Participants

The participants were FM patients, over 18 years of age, who were classified as having central sensitization syndrome.

2. Intervention

We included studies in which rTMS was applied at least 10 times to the M1 region in participants classified as having FM.

3. Comparisons

To compare the placebo effect, we included a sham group pretending to have received rTMS.

4. Outcomes

To investigate pain, quality of life (QoL) and psychosocial factors were assessed. Psychosocial factors included the health status and depression scales.

5. Types of studies

Only RCTs retrieved from four internationally used electronic databases were included. 

#### 2.2.2. Exclusion Criteria

Studies without a sham group, studies that performed rTMS in areas other than the M1, studies that did not evaluate psychosocial factors, and studies that were not RCTs were excluded.

#### 2.2.3. Literature Search Strategy

Data were collected in August 2021. Collected articles were published after rTMS was approved by the Food and Drug Administration (FDA) in 2008 [14]. As researchers with experience in meta-analysis, H.K. and S.W.L. performed independent searches. The search formula was constructed by combining the following terms: randomized controlled trials, the primary motor cortex, repetitive transcranial magnetic stimulation, and fibromyalgia.

Preidentified keywords (pain AND repetitive transcranial magnetic stimulation AND primary motor cortex AND randomized controlled trials) and the index terms were searched in all of the included databases (MEDLINE, EMBASE, CINAHL, and PEDro). Other sources were additionally searched in Google Scholar.

#### 2.2.4. Study Selection and Data Extraction

Duplicate data were removed from the documents extracted from the database in the reference management tool (EndNote 20, Thomson Reuters, New York, NY, USA). After checking the related papers by examining the titles and abstracts, the original texts of the papers selected according to the selection criteria were reviewed. Subsequently, the researchers explained why they were excluded from the literature. Finally, the selected studies were classified, and features were extracted. Data selection and extraction were independently performed by two researchers. If the data did not match, the original text was reviewed together to make a final decision.

Duplicate data were removed from documents extracted from the database in a reference management tool (EndNote 20, Thomson Reuters, New York, NY, USA). After duplicate data were removed, data were extracted according to the following procedure: First, the researchers checked the related papers by reviewing the title and abstract and reviewed the original text of the papers selected according to the selection criteria. Second, the researchers then discussed the reasons for exclusion from the literature. Finally, the selected studies were classified according to outcomes. Study selection was performed by H.K. and S.L. Data extraction was independently performed by researchers (H.K., J.J., S.P., Y.J., S.B.L. and S.W.L.) with more than 7 years of clinical and research experience in neuroscience, neurological physical therapy, and musculoskeletal physical therapy. In the case of data discrepancies during the review process, the literature was reviewed again and finally determined.

#### 2.2.5. Quality Assessment

RCTs were evaluated using the seven-item Cochrane risk of bias (RoB) tool developed by the Cochrane Bias Methods Group. The RoB was evaluated as low (+), uncertain (?), or high (-) by two researchers with experience in meta-analysis research. Items that did not match the reviews of the original text were reevaluated. Questions about evaluations that differed between the researchers were agreed upon through discussion.

### 2.3. Strategy for Data Synthesis

Data synthesis was analyzed using RevMan 5.4 (The Cochrane Collaboration, Oxford, England). Meta-analysis was performed when the same outcome variables or quantitative values of the post-test outcome variables were used, when there were three or more studies for each outcome variable, and when there were three or more studies for each outcome variable. Quantitative analysis was performed using the mean difference representing the change from baseline. However, in the study in which the change from baseline value was not described, the value of the correlation coefficient was calculated from the results using the same variables to extract the value [15]. Basically, for the effect model, a random effect model was proposed in consideration of the heterogeneity between studies [16]. 

The heterogeneity of the selected RCTs was confirmed using the chi-square test and I^2^ test provided by Cochrane. If the I^2^ values were lower than 40%, the heterogeneity was considered low; if it was 50–75%, it indicated substantial heterogeneity, and if it was 75% or greater, the heterogeneity was large [17]. Publication bias of the synthesized papers was tested using a funnel plot [18].

## 3. Results

### 3.1. Literature Search and Characteristics of the Included Randomized Clinical Trials

**Figure 1:**
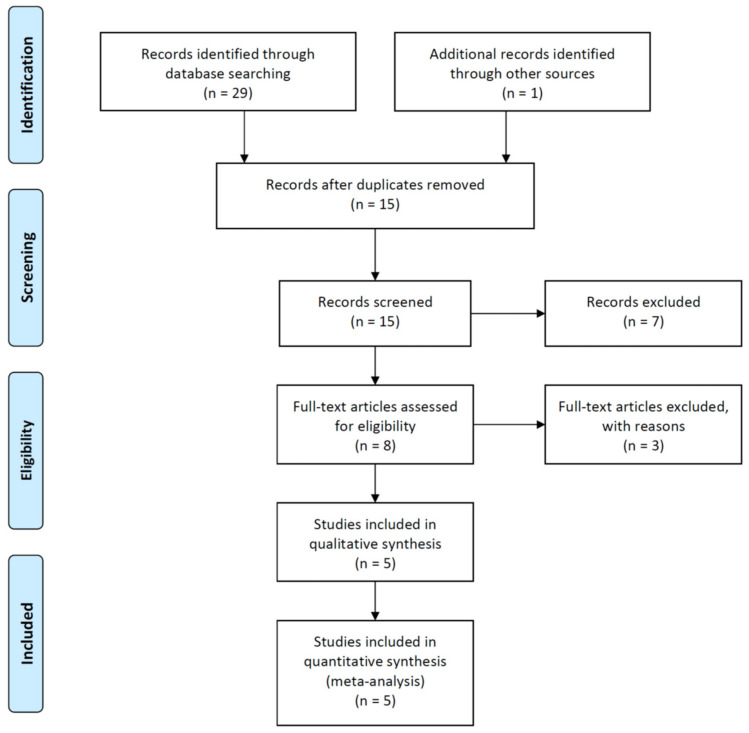
PRISMA flow diagram.

A total of 29 papers were extracted from the international database, and 1 was added through an additional search, resulting in a total of 30 papers. Fifteen papers were excluded from EndNote 20 due to duplication. Thereafter, according to the data selection criteria, two researchers reviewed the titles and abstracts, and seven papers were excluded. Among the excluded seven papers, four papers were not suitable for intervention and study design, and three papers were systematic reviews. Finally, five papers were selected, excluding three papers when reviewing full text articles [19,20,21,22,23]. Of the three excluded studies, two included tDCS without comparing rTMS and sham rTMS, and one did not include outcomes of inclusion criteria. Five selected studies were analyzed through a systematic review and meta-analysis (Figure 1).

### 3.2. Methodological Quality Assessment of the Repetitive Transcranial Magnetic Stimulation Applied to Fibromyalgia Patients

**Figure 2:**
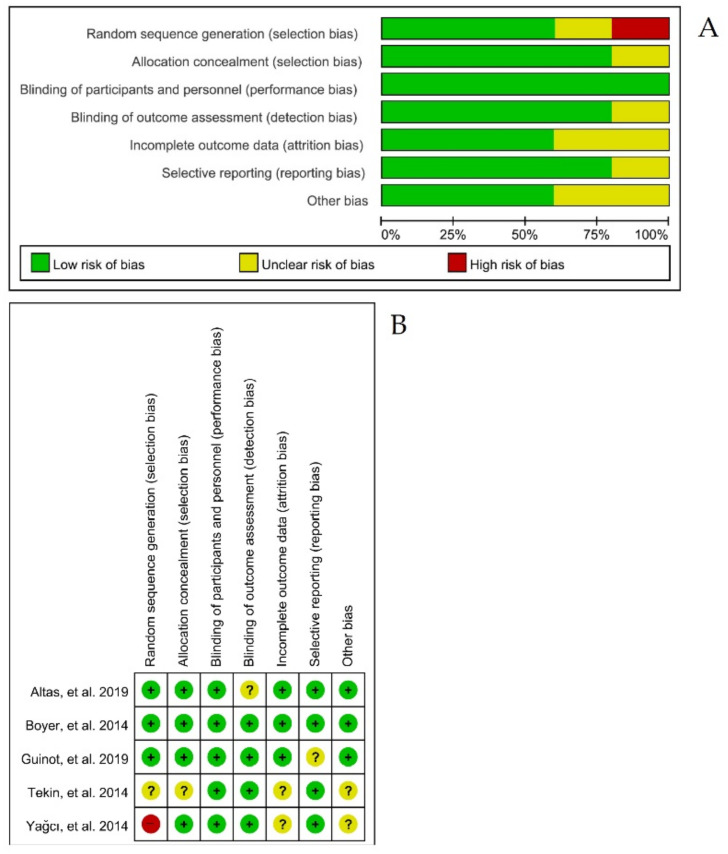
Risk of bias of the systematic review. (**A**) Risk of bias graph: review of the authors’ judgements about each risk of bias item, presented as percentages across all included studies. (**B**) Risk of bias summary: review of authors’ judgements about each risk of bias item for each included study.

A pilot test was conducted and evaluated for quality assessment in the three studies. The ‘blinding of outcome assessment’ questions required consensus among the researchers, and the concordance rate of the follow-up was 100%. Methodological quality assessments of the five experimental studies included random sequence generation (low: 3; uncertain: 1; high: 1), allocation concealment (low: 4; uncertain: 1), blinding of participants and personnel (low: 5), blinding outcome assessment (low: 4; uncertain: 1), incomplete outcome data (low: 3; uncertain: 2), selective reporting (low: 4; uncertain: 1), and other biases (low: 3; uncertain: 2) (Figure 2). The two studies classified as uncertain in other biases had differences in the baseline characteristics of participants, and the sample size calculation was not reported [24].

### 3.3. Repetitive Transcranial Magnetic Stimulation of Pain and Psychosocial Factors in Patients with Fibromyalgia

Five RCTs involving 170 patients with FM were selected for this review. Studies of rTMS applied to the M1 were included, and studies that combined other interventions were excluded. Outcome measures were macroscopically divided into pain and psychosocial factors. Pain was mainly expressed using the visual analog scale and numeric pain rating scale. Psychosocial factors included quality of life, health status, and depression. There was a significant improvement in the active rTMS group compared to the sham group, except for one study among the five selected papers (Table 1).

### 3.4. Effectiveness of Repetitive Transcranial Magnetic Stimulation on Pain

**Figure 3:**
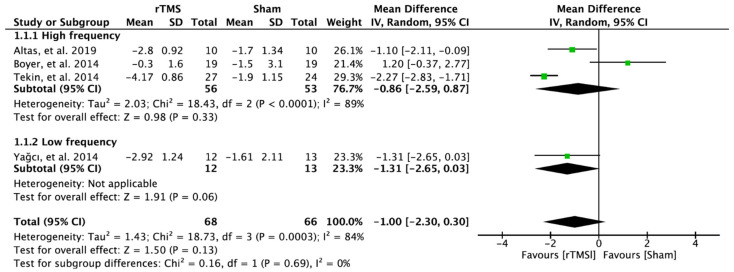
Forest plot on the effect of repetitive transcranial magnetic stimulation on pain.

In four RCTs, 134 patients with FM were evaluated for pain. There was no significant improvement in the experimental group receiving rTMS on the M1 compared to the patients receiving sham rTMS. The results analyzed through the random effect model were MD = −1.00; 95% confidence interval (CI): −2.30–0.30; heterogeneity (χ^2^ = 18.73, df = 3, I^2^ = 84%); and overall effect (Z = 1.50, *p* > 0.05) (Figure 3).

### 3.5. Effectiveness of the Repetitive Transcranial Magnetic Stimulation on Quality of Life

**Figure 4:**
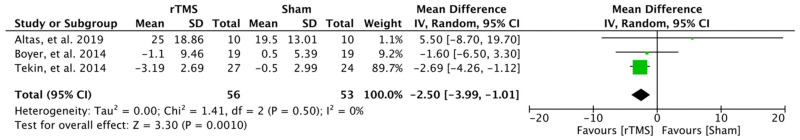
Forest plot on the effect of repetitive transcranial magnetic stimulation on quality of life.

In three RCTs, 109 patients with FM were evaluated for QoL. There was a significant improvement in the experimental group receiving rTMS on the M1 compared to the patients receiving sham rTMS. The results analyzed using the random effect model were MD = −2.50; 95% CI: −3.99–−1.01; heterogeneity (χ^2^ = 1.41, df = 2, I^2^ = 0%); and overall effect (Z = 3.30, *p* < 0.01) (Figure 4).

### 3.6. Effectiveness of the Repetitive Transcranial Magnetic Stimulation on Health Status

**Figure 5:**
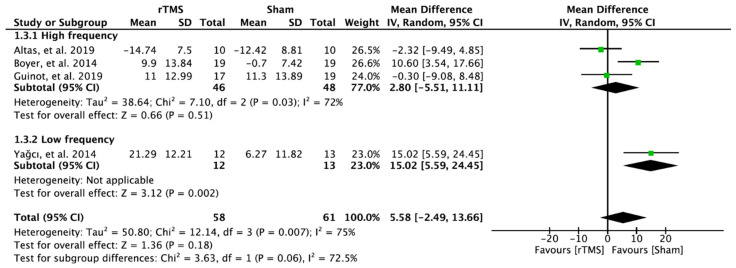
Forest plot on the effect of repetitive transcranial magnetic stimulation on health status.

In four RCTs, 119 patients with FM were evaluated using the health status. There was no significant improvement in the experimental group receiving rTMS on the M1 compared to the patients receiving sham rTMS. The results analyzed through the random effect model were MD = 5.58; 95% CI: −2.49–13.66; heterogeneity (χ^2^ = 12.14, df = 3, I^2^ = 75%); and overall effect (Z = 1.36, *p* > 0.05) (Figure 5).

### 3.7. Effectiveness of Repetitive Transcranial Magnetic Stimulation on Depression

**Figure 6:**
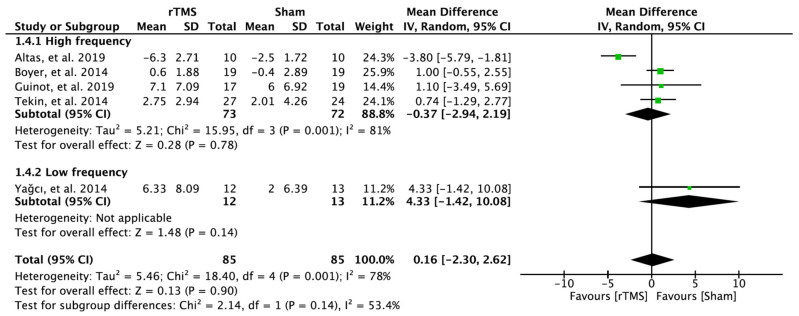
Forest plot on the effect of repetitive transcranial magnetic stimulation on depression.

In five RCTs, 170 patients with FM were evaluated for depression. There was no significant improvement in the experimental group receiving rTMS on the M1 compared to those in the sham rTMS group. The results analyzed through the random effect model were MD = 0.16; 95% CI: −2.30–2.62; heterogeneity (χ^2^ = 18.40, df = 4, I^2^ = 78%); and overall effect (Z = 0.13, *p* > 0.05) (Figure 6).

### 3.8. Publication Bias

Publication bias results via funnel plots were not analyzed because fewer than 10 studies were synthesized according to the recommendations of the Cochrane review [25].

## 4. Discussion

Our systematic review and meta-analysis synthesized and analyzed randomized controlled trials to quantify the effect of rTMS on the M1 of individuals with fibromyalgia compared with sham rTMS. Our review is the first meta-analysis to quantify the effect of limiting the application site to the primary motor cortex, unlike existing systematic reviews.

In the five studies we analyzed, as a result of applying rTMS to the M1 of individuals with FM, there was no significant improvement in all outcomes except for quality of life. When compared with sham rTMS in each of the five analyzed studies, when high frequency was applied, there was no significant difference in pain, QoL, and depression in Atlas et al. [19], and there was no significant difference in pain in Guinot et al. [21]. However, the results in Boyer et al. [20] and Tekin et al. [22] were different when high frequency was applied. Significant improvements in FIQ and QoL were shown in Boyer et al. [20], and similarly, significant improvements in pain and QoL were shown in Tekin et al. [22]. On the other hand, there was no significant difference in pain, FIQ, and BDI compared to sham rTMS in Yağcı et al. [23] when low frequency was applied. In the results, excluding Atlas et al. [19], per the rTMS guideline of Lefaucheur et al. [26], rTMS in the M1 had a significant effect on QoL in individuals with FM. Additionally, a review by Knijnik et al. [27] showed that rTMS had a better effect on quality of life than sham rTMS in FM patients one month after the initiation of treatment. However, it differed in part from previously reported systematic reviews, showing positive effects in various domains such as pain, quality of life, and depression [28]. These results were similar to but also different from our results as they analyzed application sites other than the M1. In a review by Saltychev and Laimi [11], which resulted in results similar to ours, it was judged that the application site was similar to the M1 and the dorsolateral prefrontal cortex. However, Tekin et al. [19] showed improvement in pain and depression in the sham group although the difference was not statistically significant. This was considered a placebo effect, and it has been reported that the placebo effect may actually be high in FM patients [22].

To date, there is no fully elucidated concept of the mechanism of FM. Multidisciplinary approaches such as physical therapy, cognitive therapy, education, and drugs are suggested for pain management [29]. Similarly, the physiological mechanism of rTMS is unclear, and the stimulation intensity is controversial [30]. In particular, rTMS reports different results for each application site. As in this study, studies regarding the M1 region include investigations on normalization of neural activation in the cortical motor network of post-stroke patients as well as those with chronic pain and neuropathic pain [31,32,33]. On the other hand, other areas stimulated in studies include the dorsolateral prefrontal cortex, premotor cortex, posterior parietal cortex, primary sensory cortex, secondary sensory cortex, and supplementary motor area [34,35,36,37]. 

In previous studies, many studies reported the analgesic effect by applying high-frequency rTMS to the M1 of individuals with FM [9,38,39,40,41,42]. Although only one study applied low frequency in the synthesized studies, a subgroup analysis was performed by dividing high frequency and low frequency. In frequency modulation, high-frequency rTMS activates neurons and increases cerebral perfusion, and low-frequency rTMS has the opposite mechanism [43,44]. Unlike high-frequency rTMS that has been demonstrated to some extent, there have been few studies dealing with the effects of low-frequency rTMS. An analgesic effect was reported when applied to the dorsolateral prefrontal cortex in four patients with FM [29]. In contrast, in the study applied to the dorsolateral prefrontal cortex of 28 patients with FM, there was no significant difference in pain and depression compared with the sham group [45]. Similarly, in our review, in the study to which low-frequency rTMS was applied, there was no significant difference in pain, FIQ, and BDI compared to the sham group when the M1 was stimulated [23].

In our review, we attempted to qualitatively and quantitatively incorporate RCTs of the effects of rTMS on the M1 of individuals with FM. Overall, the stimulation of the same primary motor cortex did not vary significantly depending on the frequency, but according to the results of previous studies, it is judged that high-frequency rTMS is more suitable for the M1 than low frequency. As per the guidelines [26], QoL improves significantly, but pain control is still limited. The probable mechanism of high-frequency rTMS in the M1 of individuals with FM is as follows: The temporal lobe is involved in social cognition [46], and, similarly, neural connections between the temporal region and the limbic system are elucidated [47]. In addition, the limbic system and right medial temporal cortex are involved in the control of pain-related emotional aspects in emotion modulation [48]. It has been reported that the superior temporal sulcus is involved in the functional matrix that underpins the social function of QoL [49]. Therefore, the neural connections caused by stimulation in the M1 may have an effect on the QoL by activating social cognition and emotional modulation. Although the analgesic effect was not shown in our review, considering the studies that showed the analgesic effect, the effect of pain on the emotional aspect rather than the sensory aspect should be considered. This is supported by a catastrophe that precedes the change in pain response [50] and the aforementioned neural connections.

Our review has some limitations. First, it is difficult to generalize because there are few studies conducted as RCTs. In addition, although it was not suggested in the guidelines, there was no standard protocol (session, intensity, duration, etc.), so no distinction was made except for frequency. In further studies, when psychosocial factors are considered, stimulation combined with not only the M1 but the dorsolateral prefrontal cortex is required. Furthermore, in relation to the effect on pain, it is worth evaluating the emotional aspect of pain by adding a variable for the catastrophic effect of pain.

## 5. Conclusions

Compared with sham rTMS, it could be concluded that rTMS for more than 10 sessions in the M1 of individuals with FM had an effect on QoL. Although the results differ depending on the frequency, high-frequency rTMS is recommended. In order to elucidate the analgesic effect, it is necessary to consider the effect of the emotional aspect. Future studies should include RCTs with large sample sizes.

## Figures and Tables

**Table 1:** Characteristics of included studies.

Study	Sample Size (n)	Protocol	Outcome Measures	Author’s Conclusion	Setting
Altas et al. 2019 [19]	M1 (10) Sham (10)	10 Hz, 15 sessions, 3 weeks; 90% RMT, 1200 pulses	Pain: VAS QoL: SF-36 Health status: FIQ Depression: BDI	It is effective when applied to the M1 for emotion and pain and to the DLPFC for physical function improvement.	Izmir Katip Celebi Universitesi, Izmir, Turkey
Boyer et al. 2014 [20]	M1 (19) Sham (19)	10 Hz, 14 sessions, 10 weeks; IP, 10 sessions; MP, 4 sessions	Pain: NPRS QoL: SF-36 Health status: FIQ Depression: BDI	When applied to the M1, improved QoL is associated with limbic metabolism.	La Timone University Hospital, Marseille, France
Guinot et al. 2019 [21]	M1 (17) Sham (19)	10 Hz, 16 sessions, 12 weeks; 80% RMT, 2000 pulses IP, 10 sessions; MP, 6 sessions plus 36 MT sessions	Health status: FIQ Depression: BDI	rTMS has no effect on pain control in FM patients.	Grenoble Alpes University Hospital, Grenoble, France
Tekin et al. 2014 [22]	M1 (27) Sham (24)	10 Hz, 10 sessions, 1500 pulses, 2 weeks	Pain: VAS QoL: WHOQOL Depression: MADRS	High-frequency rTMS is an alternative treatment option in FMS, effective for pain and QoL.	Si¸sli Etfal Education and Research Hospital, Istanbul, Turkey
Yağcı et al. 2014 [23]	M1 (12) Sham (13)	1 Hz (45-s interval), 10 sessions, 2 weeks; 90% RMT, 1200 pulses	Pain: VASHealth status: FIQDepression: BDI	Low-frequency rTMS may be effective in the short term, but there is no significant difference in long-term follow-up.	Medipol University Hospital, İstanbul, Turkey

DLPFC, dorsolateral prefrontal cortex; BDI, Beck Depression Inventory; FIQ, fibromyalgia impact questionnaire; FM, fibromyalgia; IP, induction phase; M1, primary motor cortex; MADRS, Montgomery−Asberg Rating Scale; MP, maintenance phase; MT, multicomponent therapy; NPRS, numeric pain rating scale; QoL, quality of life; RMT, resting motor threshold; SF-36, 36-item short-form health survey; VAS, visual analog scale; WHOQOL, World Health Quality of Life.

## Data Availability

Not applicable.
